# Antioxidant and Antiradical Activities of *Manihot esculenta* Crantz (Euphorbiaceae) Leaves and Other Selected Tropical Green Vegetables Investigated on Lipoperoxidation and Phorbol-12-myristate-13-acetate (PMA) Activated Monocytes

**DOI:** 10.3390/nu3090818

**Published:** 2011-09-16

**Authors:** Cesar N. Tsumbu, Ginette Deby-Dupont, Monique Tits, Luc Angenot, Thierry Franck, Didier Serteyn, Ange Mouithys-Mickalad

**Affiliations:** 1 Laboratory of Pharmacognosy, Department of Pharmacy, Interfaculty Centre of Drug Research (CIRM), Faculty of Medicine, Hospital Avenue 1, B36, University of Liège, Sart Tilman, 4000 Liège 1, Belgium; Email: cntsumbu@doct.ulg.ac.be (C.N.T.); M.Tits@ulg.ac.be (M.T.); L.Angenot@ulg.ac.be (L.A.); 2 Centre for Oxygen Research and Development (CORD), Institute of Chemistry B6a, University of Liège, Sart Tilman, 4000 Liège 1, Belgium; Email: cord@ulg.ac.be (G.D.-D.); t.franck@ulg.ac.be (T.F.); didier.serteyn@ulg.ac.be (D.S.); 3 Department of Clinical Sciences, Large Animal Surgery, Faculty of Veterinary Medicine, B41, University of Liège, Sart Tilman, 4000 Liège 1, Belgium

**Keywords:** monocytes, lipid peroxidation, reactive oxygen species, green vegetables, polyphenols, antioxidant

## Abstract

*Abelmoschus esculentus* (Malvaceae), *Hibiscus acetosella* (Malvaceae), *Manihot esculenta* Crantz (Euphorbiaceae) and *Pteridium aquilinum* (Dennstaedtiaceae) leaves are currently consumed as vegetables by migrants from sub-Saharan Africa living in Western Europe and by the people in the origin countries, where these plants are also used in the folk medicine. *Manihot* leaves are also eaten in Latin America and some Asian countries. This work investigated the capacity of aqueous extracts prepared from those vegetables to inhibit the peroxidation of a linoleic acid emulsion. Short chain, volatile C-compounds as markers of advanced lipid peroxidation were measured by gas chromatography by following the ethylene production. The generation of lipid hydroperoxides, was monitored by spectroscopy using *N*-*N*′-dimethyl-*p*-phenylene-diamine (DMPD). The formation of intermediate peroxyl, and other free radicals, at the initiation of the lipid peroxidation was investigated by electron spin resonance, using α-(4-pyridyl-1-oxide)-*N*-*tert*-butylnitrone as spin trap agent. The ability of the extracts to decrease the cellular production of reactive oxygen species (ROS) in “inflammation like” conditions was studied by fluorescence technique using 2′,7′-dichlorofluorescine-diacetate as fluorogenic probe, in a cell model of human monocytes (HL-60 cells) activated with phorbol ester. Overall the extracts displayed efficient concentration-dependent inhibitory effects. Their total polyphenol and flavonoid content was determined by classic colorimetric methods. An HPLC-UV/DAD analysis has clearly identified the presence of some polyphenolic compounds, which explains at least partially the inhibitions observed in our models. The role of these plants in the folk medicine by sub-Saharan peoples as well as in the prevention of oxidative stress and ROS related diseases requires further consideration.

## 1. Introduction

Monocytes and neutrophils belong to the great family of host defense cells and share similar mechanisms that consist of ingesting the bacterial material through phagocytosis and killing infectious agents by producing reactive oxygen species (ROS) upon activation of NADPH-oxidase. Furthermore, their ROS production performs other important physiological functions, for example by participating at the transduction of the cellular signal, the gene expression, the regulation of the vascular tone, *etc* [1,2,3]. 

However, an excessive ROS production can be deleterious for the cell, since ROS can attack important biomolecules, causing changes in the structure and function of enzymes, proteins and nucleotides. It is well established that ROS are involved in the long term pathogenesis of various diseases like cancer, diabetes, rheumatoid arthritis, cell aging, cardiovascular diseases, including atherosclerosis, *etc* [2,4]. Monocytes are well known to play a crucial role in the development of ROS-induced pathologies, as they can produce non-negligible amounts of ROS. 

Unsaturated fatty acids are major constituents of biomembranes, but they are particularly sensitive to oxidation. A regulated lipid peroxidation is a normal physiological and noncytotoxic process with biochemical intrinsic functions, such as the generation of prostaglandins [5]. In contrast, an increased lipid peroxidation has been found to be correlated with swelling and possible lysis of mitochondrion, microsomes, lysosomes and cells [5,6,7]. Lipid peroxides are well known to exert deleterious effects on biomembranes, for example by disturbing the lipid bilayer through inappropriate Van Der Waals associations and increasing their viscosity and osmotic fragility [6]. On the other hand, lipid peroxides react with cell proteins, modify their structure through scission, cross-linking or covalent bonds and thus, decrease their rotational and lateral mobility [5,6,7]. 

Considering the negative long-term side effects of ROS produced either by cells such as monocytes or by lipid peroxidation, modulating the ROS generation and maintaining the redox state of the cell at the required physiological level is nowadays considered as a main therapeutic target [8,9]. Polyphenols found in fruit and vegetables have gained growing interest. They have been reported to contribute to prevent or delay the onset of inflammatory diseases and other ROS related pathologies because they can develop efficient antioxidant (AOX) activities through their chemical structure [10,11] and also modulate different biochemical mechanisms within the human body [12,13,14,15,16]. In this perspective, we were interested to investigate the polyphenol content and the AOX capacity of selected green vegetables commonly consumed by migrants from Western and Central Africa living in Western Europe as well as by the people in the origin countries, *i.e*., leaves (and crosses) of *Abelmoschus esculentus* (Malvaceae), *Hibiscus acetosella* (Malvaceae), *Manihot esculenta* Crantz (Eupohorbiaceae) and *Pteridium aquilinum* (Dennstaedtiaceae). In the tropical regions, these plants grow readily even in the dry season. Among them, *Manihot* is economically the most important, because it is the basic green vegetable for people in many parts of sub-Saharan Africa such as Nigeria, Cameroon, Gabon, Democratic Republic of Congo (DRC), Uganda, Angola, *etc*., and is also consumed in Latin America, the Philippines, Indonesia, Malaysia and other Asian countries [17,18]. *Manihot* leaves (and seeds) are used in folk medicine to alleviate fever, headache, rheumatism, and hemorrhoids [19]. In Nigeria, they are also utilized in the treatment of ringworms, tumor, conjunctivitis, sores and abscesses [19]. Some literature data reported that the aqueous, methanolic and ethanolic extracts of *Manihot* leaves (and seeds) contained anthocyanins, flavonoids and other polyphenols and were found to scavenge DPPH-, hydroxyl- and superoxide free radicals, to inhibit Low Density Lipoprotein (LDL) oxidation, to chelate cupric ions and to reduce ferric ions [20,21]. Similar investigations have been carried out on *Abelmoschus esculentus* [22,23,24,25] and some *Hibiscus* varieties, mainly *Hibiscus sabdariffa* [20,25] and reported stoichiometry based AOX activities related to the polyphenol content found in the leaf and seed extracts. The leaves, flower and calyces of the variety *Hibiscus sabdariffa* are also used as diuretic, sedative, anti-scorbutic, colorectal and intestinal antiseptic as well as in heart and nerve conditions [26]. The polyphenol content of *Pteridium aquilinum* has also been investigated [27,28], but its AOX activities remain poorly elucidated [25].

Taking into account the major role of lipid peroxidation in the ROS production, originally this research consisted of designing an appropriate model to investigate the effects of the plant extracts on any of the main steps of lipid peroxidation, beginning with markers of an advanced lipid peroxidation, and investigating back along the pathway of lipid peroxidation to its initiation, where transient free radicals are produced. Short chain volatile *C*-compounds like pentane and ethylene are markers of an advanced lipid peroxidation [29]. In our model, their formation was analyzed with a gas chromatography (GC) technique by following the ethylene production. Lipid hydroperoxides are generated before the volatile compounds [29]. Their formation was investigated with spectroscopy technique using *N*-*N*′-dimethyl-*p*-phenylene-diamine (DMPD). The generation of transient free radicals characterizes the initiation of a lipid peroxidation [29]. The formation of these intermediate compounds was studied with Electron Spin Resonance technique (ESR). The second set of experiments was a model of ROS production based on the oxidant activities of human monocytes activated with phorbol-12-myristate-13-acetate (PMA), that we designed to study the effect of the plant extracts on the cellular ROS production in inflammation-like conditions, using a fluorescence technique with 2′,7′-dichlorofluorescine-diacetate (DCFH-DA) as fluorogenic probe. Compared to neutrophils, monocytes have low level of myeloperoxidase, which is a powerful oxidant enzyme mainly occurring in the defense cells, particularly in neutrophils [30]. Consequently, the ROS amount produced by monocytes could be quite weak. Therefore, horseradish peroxidase (HRP) was used as reaction enhancer to increase the ROS level and allow easy comparisons of the inhibitory effects displayed by the extracts being studied. To reproduce at best the usual culinary processing and to analyze its eventual effect on the AOX capacity of the tested plants, samples of each plant extract were boiled and tested in parallel to not heated samples. The amount of total polyphenol, non-tannin polyphenol and flavonoid contained in the dry plant powders was analyzed using classic spectroscopy techniques. To identify some of the polyphenols contained in the plant extracts, an HPLC-UV/DAD analysis was undertaken, using some standard compounds with well-known AOX activities as reference.

## 2. Experimental Section

### 2.1. Chemicals and Reagents

Sodium carbonate, sodium dihydrogenphosphate, di-sodium hydrogenphosphate, hydrochloric acid, iron(II) sulfate heptahydrate, ammonium iron(II) sulfate hexahydrate, vitamin C, ethyl acetate, acetone, methanol and ethanol were of analytical grade from Merck VWR (Leuven, Belgium). Linoleic acid, phorbol-12-myristate-13-acetate (PMA), polyphenol standards, 3-[(3-cholamidopropyl)-dimethylammonio]-1-propanesulfonate (CHAPS), phosphomolybdo-phosphotungstic reagent (Folin-Ciocalteu reagent), as well as glacial acetic acid, sheep skin powder, aluminium chlorid, anhydrous sodium sulfate, hexamethylentetramine, *N*-*N*′-dimethyl-*p*-phenylene diamine (DMPD) and α-(4-pyridyl-1-oxide)-*N*-*tert*-butylnitrone (POBN) were from Sigma (Bornem, Belgium). Chelex (200–400 mesh, sodium form) was from BioRad (Belgium); chloroform was from Acros (Geel, Belgium). 2′,7′-dichlorofluorescine-diacetate (DCFH-DA) was purchased from Eastman Kodak (Rochester, NY, USA), Trypan blue was from ICN Biomedicals, Inc (Cleveland, OH, USA), and horseradish peroxidase (HRP) was obtained from Roche (Mannheim, Germany). Water was treated in a Milli-Q water ultra purification system (Easy Pure Purification System).

### 2.2. Vegetal Material

*Abelmoschus*, *Hibiscus*, *Manihot* young leaves and *Pteridium* crosses and leaves were gathered in Kisantu, province Bas-Congo (DRC) in March–April 2007, and authenticated by the National Institute for Research in Agronomics (INRA), University of Kinshasa, DRC. Sample specimens bearing authentic voucher numbers were provided to the affiliation laboratory of the research team. The plant parts were shaded dried until their weight became stable, then ground and sieved at 180 µm particle size. The recovered powder was loaded into hermetic and opaque flasks and stored at room temperature out of light until further preparations.

### 2.3. Preparation of Aqueous Extracts and Samples for Biochemical Assays

The powder (100 g) was permanently stirred (300 rpm) for 6 h at room temperature in 500 mL ultra pure water until filtration and lyophilization (24 h). The extracts were kept in hermetic and opaque flasks at −22 °C. Before each assay, two measures of each extract were dissolved in ultra pure water; one of them was boiled for 45 min. All steps were performed in the dark. The extraction yields were *Pteridium* 7.9% > *Manihot* 7.4% > *Hibiscu*s 7.3% > *Abelmoschus* 6.9%.

### 2.4. Study on Lipid Peroxidation Model

#### 2.4.1. Preparation of the Linoleic Acid Emulsion

Linoleic acid (0.64 mM) and 200 mg CHAPS were mixed in 50 mL chelexed phosphate buffer (pH 7.4) and gently shaken for 5 min at room temperature.

#### 2.4.2. Measurement of the Ethylene Produced (Gas Chromatography Technique)

The experiment was based on the procedure described by Beyer *et al*. [31]. Two hundred microliters of plant extract adjusted to 10, 50 and 100 µg/mL final concentration, one milliliter of linoleic acid emulsion 0.64 mM, 200 µL of vitamin C 1 mM and 580 µL of phosphate buffer pH 7.4 were loaded into flasks. Iron(II) 5 mM (20 µL) was added before the flasks were hermetically sealed and kept at 37 °C overnight in the dark. Ten microliters of the upper gaseous phase produced in the flask were manually injected in the column of a Varian 450 GC apparatus. One flask was loaded without plant extract and was set as 100% ethylene production (control). The GC parameters were: Porapak T column (1 m length; ID 1/8 inch; supplied by Supelco, Belgium) at 160 °C. Nitrogen was used as gas carrier (30 mL/min). Flame ionization detector (FID) and injector temperatures detector were both set at 200 °C. The driving software was the Varian “Galaxy Chromatography data system”. 

#### 2.4.3. Measurement of the Lipid Hydroperoxides Produced (UV-Visible Assay with DMPD)

The assay was based on the method described by Deby *et al.* [32], with light adaptations. One hundred microliters Vitamin C (1 mM), 90 µL phosphate buffer (pH 7.4) and 100 µL of extract solutions adjusted to respectively 5, 10, 25, 50, 75 and 100 µg/mL final concentration, were added to 700 µL linoleic acid emulsion (0.45 mM). Ten microliters Fe(II) 5 mM were loaded as last before the tube was hermetically closed and stored for 4.5 h at 37 °C in the dark. One tube was loaded without plant extract and was set as control for the lipid peroxidation (100% production of lipid hydroperoxides). Before centrifugation (400× *g*, 10 min, 20 °C), 2 mL chloroform were added to extract the organic phase. The aqueous layer was discarded and the organic phase was immediately evaporated under a flow of nitrogen gas. To monitor the peroxidation products, 300 µL ethanol, 3 mL DMPD solution (2.2 µM DMPD in chloroform/acetic acid/ultra pure water 5:5:1 v/v/v) and as last 50 µL Fe(III) 0.128 µM final concentration were loaded before the absorbance was read using ethanol as blank (Hewlett Packard Spectrophometer, 517 nm).

#### 2.4.4. Measurement of the Transient Free Radicals Produced by Lipid Peroxidation (ESR Technique)

This study was performed according to the method described by Mouithys-Mickalad *et al.* [33]. Plant extracts (100 µL) were adjusted to 10, 50 and 100 µg/mL final concentration and added to 500 µL linoleic acid emulsion (0.32 mM) before addition of 100 µL vitamin C (1 mM) and 100 µL POBN (50 mM). One flask of the emulsion received no plant extract and was used as control for the formation of intermediate free radicals (100% production of transient free radicals). Each tube volume was completed to 1 mL total volume with chelexed phosphate buffer (pH 7.4) and 10 µL 5 × 10^−5^ M Fe(II) that was added at last. The tube was stored in the incubator at 37 °C in the dark for 2 h and the content transferred in an ESR quartz flat cell which was put into the cavity of the ESR spectrometer for analysis. The measurements were carried out at room temperature with a Bruker spectrometer (Bruker, Karlsruhe, Germany), operating at X-band frequency (9.8 GHz) and at a microwave power of 20 mW. The instrumental settings were: 100 KHz modulation frequency, 1.012 G modulation amplitude, 3480 G magnetic field centre; receiver gain was 2 × 10^4^. The sweep width was ±50 G and the number scan was 1. The hyperfine splitting constants were measured from the experimental spectra using a Bruker Win-Simfonia program running under Microsoft Windows.

### 2.5. Study on Cell Model

#### 2.5.1. Cell Culture

Human promyelocytic leukemia cells (HL-60) were obtained from the American Type Culture Collection (ACCT, USA) and cultured in Iscove’s Modified Dulbecco’s Medium (IMDM) supplemented with 20% (v/v) fetal calf serum, 100 U/mL penicillin/streptomycin, 1.25 mg/mL amphotericin B, and 2 g/L NaHCO_3_ in 50 mL flasks at 37 °C in a 5% CO_2_ humidified atmosphere. The cells were cultured and fed two to three times per week to maintain a log phase growth and once a week, they were centrifuged and re-suspended in fresh IMDM. Before each experiment, cells were counted with Burker’ cell (Briare, France) to reach the cell density required for the experiments, *i.e*., 1 × 10^6^ cells/mL.

#### 2.5.2. Cell Viability Assay

Cells (10^6^/mL HBSS buffer) were incubated with 10 µL of not heated extract solutions at the final concentration of 100 μg/mL at 37 °C for 45 min in the dark. The cell viability was checked using an exclusion test with Trypan blue [34]. For the control cells, 10 µL ultra pure water were used instead of the extract solutions.

#### 2.5.3. Measurement of the ROS Produced by PMA-Activated HL-60 Monocytes (Fluorescence Technique with Non Fluorescent DCFH-DA)

This experiment was based on the method described by Amado *et al*. [35] with adaptations. One million HL-60 monocytes were incubated at 37 °C in the dark with 41 µM non-fluorescent DCFH-DA for 45 min in 24-well microtiter plates. The content of each well was transferred into a 5 mL-tube for centrifugation (300× *g*) for 10 min at 37 °C. The recovered cell pellet was diluted with 500 µL HBSS buffer and transferred into wells containing 450 µL HBSS. Ten microliters of extract solution at final concentrations of 10, 50 and 100 µg/mL, 10 µL HRP (30 µg/mL) and as last 30 µL PMA (0.486 µM) were added to a volume of 950 µL cell suspension. In each assay, three wells were loaded without plant extract and were taken as control for the ROS production (100% ROS induced fluorescence). To measure the basic ROS production of the cells at the absence of activation, three other wells without plant extract received no PMA (NA, not activated). The fluorescence produced was measured for 30 min at 37 °C on a Fluoreskan (Fluoreskan Ascent FL, Fischer Scientific, Tournai, Belgium).

### 2.6. Estimation of the Content of Phytochemical Compounds

#### 2.6.1. Spectroscopy Based Estimation of Polyphenols and Tannins

##### 2.6.1.1. Extraction Procedure 

The powder (1.5 g) was permanently stirred in 250 mL ultra pure water and simultaneously heated for 30 min at 60 °C under backward flow until filtration on a paper filter (125 mm). Fifty milliliters of the supernatant were removed before the filtrate was diluted in ultra pure water (1/4 v/v). 

##### 2.6.1.2. Estimation Technique

Total polyphenols were estimated according to the Folin-Ciocalteu spectroscopy based method [36]. An aliquot of 200 µL of the sample prepared above was mixed with 100 µL of Folin-Ciocalteu reagent in 1 ml ultra pure water and vortexed. 1.2 mL of an aqueous solution of Na_2_CO_3_ (200 g/L) were added and the mixture was allowed to stand for 30 min at room temperature in the darkness before the absorbance was measured. To determine the tannin content, 100 mg of sheepskin powder were dissolved in 10 mL sample and shaken for 1 h. The volume of the resulting filtrate was increased with ultra pure water to 1:4 v/v. The absorbance was read at 760 nm, using pyrogallol as control and water as blank (Spectophotometer UVIKON 922). The results, expressed as g pyrogallol equivalent/100 g dry plant powder, were calculated according to the formulae: total polyphenol = [(62.5 × A_1_) × m_2_]/(A_3_ × m_2_), non-tannin polyphenol = [(62.5 × A_2_) × m_2_]/(A_3_ × m_1_) and tannins = [62.5 × (A_1_ − A_2_) × m_2_]/(A_3_ × m_1_), whereby A_1_, A_2_ and A_3_ are the absorbance values of total polyphenol (A_1_), non-tannin polyphenol (A_2_) and pyrogallol (A_3_), m_1_ and m_2_ are the mass (in gram) of respectively the plant powder (m_1_) and pyrogallol (m_2_) that were weighed for this determination. 

#### 2.6.2. Colorimetry Based Estimation of Flavonoids

##### 2.6.2.1. Extraction Procedure

Plant powder (200 mg) was dissolved in a mixture of 35 mM hexamethylenetetramine, acetone and hydrochloric acid (1:20:2 v/v/v), boiled for 30 min under backward flow and filtered through absorbent cotton. The filter and the residual plant marc were equally boiled for 10 min in 40 mL acetone and filtered. The volume resulting from both filtrate fractions was completed to 100 mL with acetone, mixed with ultra pure water and ethyl acetate to 1:1:2.25 v/v/v, and then submitted to extraction on 10 g anhydrous sodium sulfate; the volume of the ultimate filtrate was completed to 50 mL with ethyl acetate. 

##### 2.6.2.2. Estimation Technique

The content of flavonoid was estimated by the AlCl_3_ technique according to the method described in the European Pharmacopeia [37]. Glacial acetic acid and methanol were mixed to 1:19 v/v. Four hundred microliters of the plant sample prepared above, 40 µL AlCl_3_ and 560 µL of the glacial acetic acid-methanol mixture were loaded into vials that were allowed to stand for 30 min at room temperature out of light before the absorbance was read at 425 nm (Spectophotometer UVIKON 922), using hyperoside as control and water as blank. The results, expressed as g hyperoside equivalent/100 g dry plant powder, were calculated using the formula: (A × 1.25)/m, whereby A is the absorbance value and m the mass (in gram) of the extract weighed for this determination. 

#### 2.6.3. HPLC-UV/DAD Identification of Some Compounds Contained in the Plant Extracts

The samples were extracted from 1 g plant powder boiled for 5 min in 10 mL methanol at 60 °C under backward flow and filtered on a Whatman paper filter. One milligram of respectively rutin, hyperoside, cafeic, chlorogenic and rosmarinic acids of HPLC analytical grad was dissolved in 10 mL methanol and gently shaken. The analysis was carried out at 25 °C by an “Agilent 1100” HPLC chain connected to a diode array detector (DAD). All samples and standards were filtered through a 0.45 µm pore size syringe-driven filter before 20 µL of each one were injected into the HPLC-UV/DAD system. The separation was carried out using an Hypersil ODS column (4 mm × 250 mm) with a nonlinear gradient of acetonitril (solvent A) and 0.05% trifluoroacetic acid in ultra pure water (solvent B) in the following composition: T_0_: 0% A, 100% B; T_1_: 3% A, 97% B; T_45_: 40% A, 60% B; T_46_: 0% A, 100% B and T_60_ stop. The time (T) is expressed in minutes. The compounds were eluted at a flow rate of 1 mL/min and detected with UV-DAD. The UV spectra of elution peaks were recorded in the range from 250 to 340 nm and the chromatograms were monitored at 280 and 340 nm. The identification was based on the retention time and the absorption spectra in comparison to the references and the data available in the database of the system. 

### 2.7. Statistical Analysis

Each concentration was tested in triplicate in each assay. The fluorescence, ESR and GC assays were carried out two times, the DMPD technique three times. All results were expressed as mean values ± standard deviation (SD). The statistical analysis was performed with GraphPad Instat 3.05 (GraphPad Software, San Diego California, USA). The results were analyzed using one-way analysis of variance (ANOVA); a multiple comparison of all data was performed using the “Student-Newman-Keuls Multiple Comparisons Test”, particularly focusing on two comparisons within each assay: “each sample *vs.* control”, and for the same extract at the same concentration, “boiled sample *vs.* not heated sample”. The IC_50_ values to the DMPD assay were calculated with GraphPad Prism 5.0 under application of the function “log (inhibitor) *vs*. normalized response-variable slope” after converting the concentrations into their decimal logarithm. 

## 3. Results

### 3.1. Effect of the Extracts on the Ethylene Production (GC Assay)

**Figure 1:**
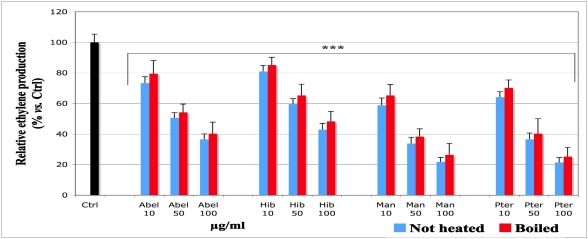
Effect of the aqueous extracts of *Abelmoschus esculentus*, *Hibiscus acetosella*, *Manihot esculenta* and *Pteridium aquilinum* on the ethylene amount, produced by an advanced lipid peroxidation. Solutions of plant extracts at the final concentrations given under the histograms, 1 mM vitamin C and 20 µL Fe(II) were loaded to 1 mL emulsion of linoleic acid 0.64 mM in phosphate buffer pH 7.4. The flasks were hermetically sealed and kept overnight at 37 °C in the dark. Ten microliters of the gaseous phase were manually injected in the column of a GC apparatus. For the control, *i.e.*, linoleic emulsion without plant extracts set as 100% ethylene production (Ctrl, black histogram), an equivalent volume of the buffer was used instead of the plant extracts. The values are presented as average of the relative percentage *vs.* Ctrl ± standard deviation (SD). *** *p*-value < 0.001 for all samples *vs.* Ctrl. For the same extract at the same concentration, not heated *vs.* boiled samples: no significance (*p* > 0.05 overall). Abel = *Abelmoschus*, Hib = *Hibiscus*, Man = *Manihot*, Pter = *Pteridium*.

The ethylene production in the absence of the plant extracts was set as control (Figure 1, black histogram, Ctrl, *i.e.*, 100% ethylene production). The addition of the extracts at different final concentrations inhibited significantly the ethylene production in a concentration-dependent relationship. For the same extract at the same concentration, the levels of the residual ethylene production were moderately higher for the boiled than for the not heated samples. However, the differences were generally not significant. The following inhibitory efficiency was observed: *Manihot* > *Pteridium* > *Abelmoschus* > *Hibiscus*. 

### 3.2. Effect of the Extracts on the Production of Lipid Hydroperoxides (UV Visible Assay with DMPD)

**Figure 2:**
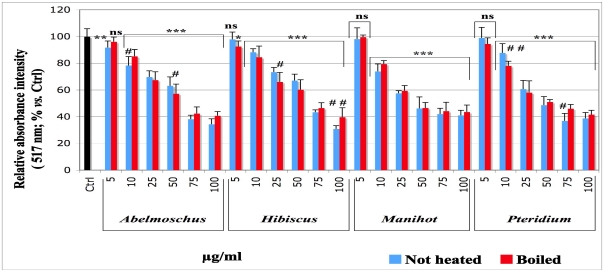
Effect of the aqueous extracts of *Abelmoschus esculentus*, *Hibiscus acetosella*, *Manihot esculenta* and *Pteridium aquilinum* on the formation of lipid hydroperoxides by lipid peroxidation. Vitamin C (1 mM), 90 µL phosphate buffer (pH 7.4), extract solutions at the final concentration given under the histograms, and Fe(II) 5 mM were loaded to 700 µL emulsion of linoleic acid 0.45 mM. The tubes were hermetically closed and stored for 4.5 h at 37 °C in the dark. Two milliliters of chloroform were added before centrifugation (400× *g*, 10 min, 20 °C). The aqueous layer was discarded and the organic phase was evaporated with nitrogen gas. The peroxidation products were monitored by adding 300 µL ethanol, 3 mL DMPD solution and 50 µL Fe(III) into the tubes. The absorbance was immediately read at 517 nm using ethanol as blank (Hewlett Packard Spectrophometer). The control (Ctrl, black histogram; set as 100% production of lipid hydroperoxides) consisted of the complete lipoperoxidation system with an equivalent volume of phosphate buffer instead of the plant extracts. The values are presented as average of relative percentage *vs.* Ctrl, ± standard deviation (SD). Samples *vs.* Ctrl: ns no significance; * *p* < 0.05; ** *p* < 0.01; *** *p* < 0.001. For the same extract at the same concentration, not heated *vs.* boiled samples: ^#^ *p* < 0.05; ^##^ *p* < 0.01; all other comparisons (not shown): no significance.

The results are shown on Figure 2. The intensity of the absorbance signal at the absence of the tested plant extracts was set as control (Ctrl, black histogram, 100% intensity). The addition of the plant extracts at a final concentration of 5 µg/mL displayed no noticeable inhibitory effects on the production of lipid hydroperoxides, whatever the plant. In contrast, the addition of final extract concentrations higher than 5 µg/mL decreased significantly (* *p* < 0.001) the intensity of the absorbance in a dose-dependent manner. The data obtained with boiled and not heated samples for the same extract at the same concentration were generally not significantly different (*p* > 0.05), except for *Abelmoschus* at 10 and 50 µg/mL, *Hibiscus* at 25 µg/mL and *Pteridium* at 75 µg/mL (*p* < 0.05), as well as for *Pteridium* at 10 µg/mL (*p* < 0.01), *Abelmoschus* at 75 µg/mL and *Hibiscus* at 100 µg/mL (*p* < 0.01). The IC_50_ values for boiled and not heated samples (Table 1) showed the following order of inhibitory efficiency: *Manihot* > *Pteridium* > *Abel > Hibiscus* without great differences from one plant to another. 

**Table 1:** IC_50_ values (µg/mL) for the inhibition of the production of lipid hydroperoxides in the UV-visible assay with DMPD. Values are expressed as average ± standard deviation (SD); *n* = 9.

Plant	Not heated samples	Boiled samples
*Abelmoschus esculentus*	61.25 ± 2.6	57.68 ± 9.3
*Hibiscus acetosella*	66.34 ± 8.19	63.37 ± 1.8
*Manihot esculenta* C.	55.08 ± 9.83	49.32 ± 10.23
*Pteridium aquilinum*	55.24 ± 4.04	49.61 ± 5.67

### 3.3. ESR Spin Trap Study: Radical Scavenging Effect of the Extracts

**Figure 3:**
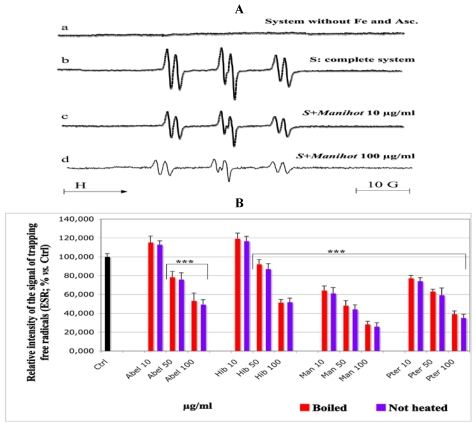
Effect of the aqueous extracts of the tested plants on the formation of transient free radicals by lipid peroxidation (ESR technique). One hundred microliters of solutions of the plant extracts at the final concentrations given under the histograms were charged into 500 µL of emulsion of the linoleic acid 0.32 mM. Vitamin C 1 mM and 50 mM POBN were added. The volume was completed to 1 mL with chelexed phosphate buffer (pH 7.4) and 5 × 10^−5^ M Fe(II). The tubes were hermetically closed and stored at 37 °C in the dark for 2 h. Then, the content was transferred into an ESR quartz flat cell for monitoring the formation of intermediate free radicals with an ESR spectrometer. No spin trapping signal was observed at the absence of the Fe/Vit C redox system (Figure 3**A**, spectrum a). The addition of the Fe(II)/Vit C system initiated the lipid peroxidation and led to a signal of spin trapping with a high intensity (Figure 3**A**, spectrum b). This value, measured on the complete system at the absence of the plant extracts, was set as control (Figure 3**B**, Ctrl, black histogram, 100% signal intensity). Except *Abel* and *Hib* at 10 µg/mL which enhanced the signal, the addition of the extract solutions at increasing concentrations decreased the intensity of the spin trapping signal (Figure 3**A**, spectrum c, d and Figure 3**B**). Samples *vs.* Ctrl: ** *p* < 0.01; ***p < 0.001. The same extract at the same concentration, not heated *vs.* boiled sample: no significance. Abel = *Abelmoschus*, Hib = *Hibiscus*, Man = *Manihot* and Pter = *Pteridium*.

The results are summarized in Figure 3. In the absence of the ascorbate/Fe(II) system, the lipid peroxidation did not start (Figure 3A, spectrum a). In contrast, the attack of the linoleic acid emulsion by the triggering system (ascorbate/Fe(II)) in presence of POBN led to an ESR spectrum of high intensity (Figure 3A, spectrum b), which characterizes the trapping of free radicals by the nitrone spin trap agent [30]. Compared to the control (Figure 3A, spectrum b; Figure 3B, Ctrl, black histogram) the addition of extract solutions at increasing final concentrations (10, 50 and 100 µg/mL) resulted in a decrease of the intensity of the ESR spectra in a concentration dependent manner (Figure 3A, spectra c, d and Figure 3B, sample histograms *vs*. Ctrl). All extracts showed extremely significant inhibitory effects *vs*. control (Figure 3B, *** *p* < 0.001) depending on the concentration and the plant, except *Abelmoschus* and *Hibiscus* at 10 µg/mL, which increased the formation of free radicals, compared to the control. For the same extract at the same concentration, not heated samples decreased the formation of free radicals more than the boiled ones, but the difference remained not significant *(p* > 0.05). 

### 3.4. Effect of the Plant Extracts on the Cell Viability

**Figure 4:**
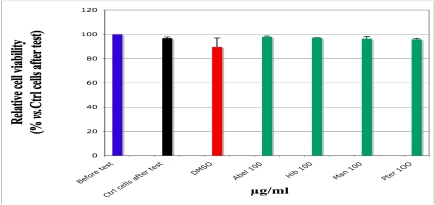
Viability of monocytes in presence of aqueous extracts of *Abelmoschus esculentus*, *Hibiscus acetosella*, *Manihot esculenta* and *Pteridium aquilinum*, measured by exclusion test with Trypan blue. The cells (10^6^/mL HBSS) were incubated for 45 min with not heated extract solutions at the final concentration of 100 μg/mL at 37 °C, in the dark. The cell population before test (blue histogram) was set as reference (100%). For the control cells (black histogram), ultra pure water was used instead of the extract solutions. The values are presented as average of the relative percentage *vs.* Ctrl ± standard deviation (SD). Abel = *Abelmoschus*, Hib = *Hibiscus*, Man = *Manihot* and Pter = *Pteridium*.

The results of cell viability are presented on Figure 4. At the highest extract concentration that was applied in our assays (100 µg/mL), the extracts of vegetables caused no cell toxicity and there was no significant difference of viability between cells incubated with plant extracts and those without extract solutions (control cells). 

### 3.5. Effect of the Extracts of Vegetables on the ROS-Induced Fluorescence

**Figure 5:**
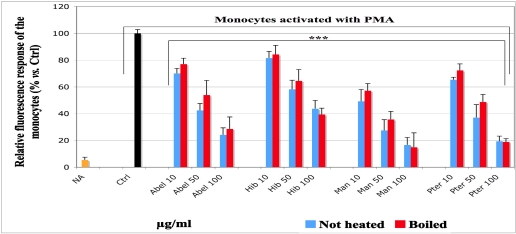
Effect of the aqueous extracts of *Abelmoschus esculentus*, *Hibiscus acetosella*, *Manihot esculenta* and *Pteridium aquilinum* on the ROS production by PMA activated HL-60 monocytes. Monocytes (10^6^/mL) were incubated for 45 min with 41 µM non-fluorescent DCFH-DA probe in the dark, centrifuged (300× *g*) for 10 min at 37 °C and diluted in HBSS buffer. Ten microliters extract solution at the given final concentrations, 300 ng HRP and 0.486 µM PMA were successively added to 950 µL cell suspension. The fluorescence due to the reaction of the desacetylated fluorogenic probe with the ROS produced by the monocytes activated with PMA was measured for 30 min at 37 °C with a Fluoreskan. The fluorescence displayed by activated cells at the absence of plant extracts was set as control (Ctrl, black histogram; 100% fluorescence). The values are presented as average of the relative percentage *vs.* Ctrl ± standard deviation (SD). Samples *vs.* Ctrl: *** *p* < 0.001. For the same extract at the same concentration: overall values no significance. Abel = *Abelmoschus*, Hib = *Hibiscus*, Man = *Manihot* and Pter = *Pteridium*. NA: non-activated.

The height of the histograms in Figure 5 shows the intensity of the fluorescence resulting from the reaction between DCF and the ROS produced by the activated monocytes (HL-60 cells). The fluorescence observed in the absence of the extracts was taken as 100% (black histogram; Ctrl). The addition of extract solutions at increasing final concentrations (10, 50 and 100 µg/mL) resulted in decrease of the ROS-induced fluorescence depending on the concentration. For all extracts, the intensity of the fluorescence due to the level of the residual ROS production showed an extreme significant difference *vs*. control (*p* < 0.001). *Manihot* was the most efficient, followed by *Pteridium*, *Abelmoschus* and *Hibiscus*. The not heated samples displayed slightly more inhibitory efficiency than the boiled ones. However, for the same extract at the same concentration, no significant difference was found between boiled and not heated samples (*p* > 0.05). 

### 3.6. Phytochemical Analysis

The polyphenol and flavonoid content of the plants is presented on Table 2. *Pteridium* was shown to have the highest total polyphenol amount, but the elimination of tannins revealed that 73% of its total polyphenol were tannins, whereas *Abelmoschus* and *Hibiscus* had respectively 40 and 36% of tannins in the total polyphenol amount (for *Manihot*: content not determined). *Hibiscus* and *Manihot* contained nearly the same amount of flavonoids, whereas *Pteridium* had the weakest one (Table 2).

**Table 2:** Total polyphenol and flavonoid content estimated with spectroscopic methods.

Sample	*Abelmoschus*	*Hibiscus*	*Manihot*	*Pteridium*
Total polyphenol ^a^	1.48	1.73	ND	6.74
Total flavonoid ^b^	ND	1.54	1.53	0.58

^a^ Total polyphenols were measured with the Folin-Ciocalteu method and expressed as % pyrogallol weight-equivalent, *i.e.*, g/100 g of the dry plant powder; ^b^ Total flavonoids were determined by the aluminium chloride (AlCl_3_) colorimetric method and expressed as % hyperoside weight-equivalent, *i.e.*, g/100 g of the dry plant powder. ND: not determined.

**Figure 6:**
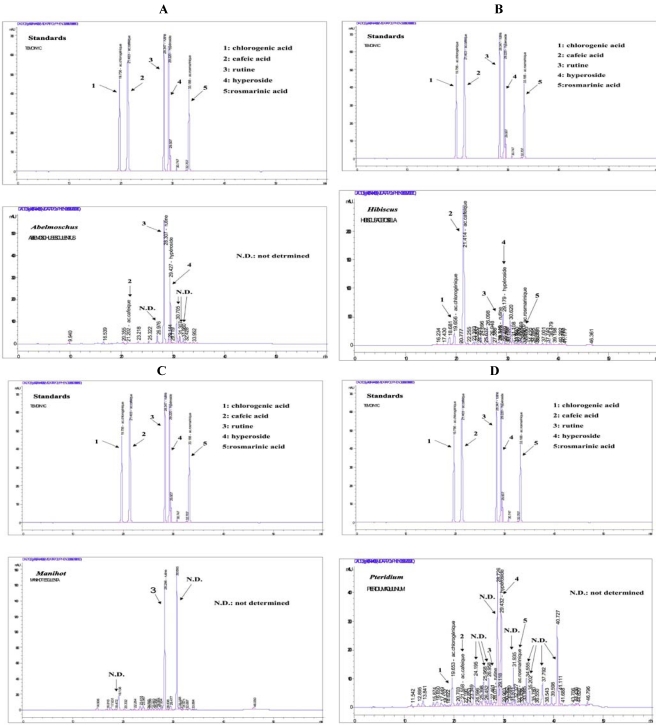
HPLC-UV/DAD chromatograms of the tested plants. The chromatograms were obtained from methanolic extracts of the dry plant powders with a RP HPLC-UV/DAD analysis on an ODS column. The elution time is given in minutes (horizontal axis). On the y-axis, the height of the elution peak, expressed in arbitrary milli-unities (mAU) corresponds to the concentration (data not given) of the eluted compound in the sample analyzed. From **A** to **D**: Upper part = the standards used as reference; lower part = the plant analyzed: **A** = *Abelmoschus*, **B** = *Hibiscus*, **C** = *Manihot*, **D** = *Pteridium*.

Among the standard compounds that we utilized as reference for the HPLC analysis, some ones were identified in some samples and are presented on Figure 6A–D. *Abelmoschus* was recognized to contain noticeable amounts of rutin, hyperoside, and some cafeic acid (Figure 6A). *Hibiscus* had an important quantity of cafeic acid, a noticeable amount of hyperoside as well as some chlorogenic acid, rosmarinic acid and rutin (Figure 6B). *Manihot* contained two main compounds and weak quantities of some other ones, but taking into account the standard compounds that we used as reference, only rutin could be clearly identified (Figure 6C). *Pteridium* contained all the standards in different amounts, principally hyperoside. However, many of its components were not identical with the standards that we used as reference and thus, could not be identified (Figure 6D). 

## 4. Discussion

In the traditional use, the tested green vegetables are previously chopped, immersed in water, roasted, then crushed and cooked in boiling water for 30 to 45 min before consumption. Such processing eliminates cyanosids and ptaquilosids, well known toxic compounds of *Manihot* and *Pteridium*, or decreases them under a non-toxic level. Under these usual conditions, the consumption provokes neither digestive problems nor allergic symptoms. The results obtained with our cellular model of viability are in agreement with this traditional use and literature data [38,39]. 

Several methods have been developed to extract the antioxidant (AOX) fraction of plant and food material. The extraction yield and the activities of the obtained extracts depend on many factors, such as the solvent used and its polarity, temperature, pH, the plant part, material preparation and the presence of hydrophilic and/or lipophilic compounds. In this work, we determined the polyphenol content with samples prepared on specific procedures appropriated to these compounds [36,37]. The plant powders were shown to contain different amounts of total polyphenol and flavonoid, depending on the plant (Table 1). The spectroscopy analysis with Folin-Ciocalteu reagent showed that *Pteridium* had the highest amount of tannins. Tannins might also intervene in the AOX activities that we observed with *Pteridium* samples. Indeed, tannins are found in many plant foods including walnuts and pomegranates. At moderate levels, these flavanol polymers are non-toxic and develop efficient AOX capacity [40]. For instance, ellagic acid released from hydrolysable tannins in the small intestine is a compound with well-known AOX efficiency. Furthermore, tannins are metabolized by gut bacteria into urolithins which may have further health benefits [41].

As shown in Figure 6A–D, a preliminary HPLC separation carried out on methanolic plant extracts has permitted identification of some polyphenolic compounds in the samples. Other compounds, whose standards were not put in the analysis set, still remain to be identified by further analysis. However, the identified compounds sufficiently explain at least partially the inhibition of oxidation that we observed, since they complete some classic criteria, such as the presence of hydroxyl groups around the aromatic rings that is required by polyphenols to enable liberation of electrons and efficiently inhibit oxidation [10,11]. 

For the biochemical experiments, we were interested to mimic the processing usually carried out by the migrants from sub-Saharan Africa living in Western Europe and by the people in the origin countries, consisting of cooking these vegetables in boiling water. Thus, despite the eventual differences in yield and biological activities of the extracts obtained from the different extraction procedures, we have used aqueous extracts for the lipid and cellular models.

ROS produced within the cell might be harmful, for example by attacking polyunsaturated fatty acids and damaging the membrane lipid bilayer [5,6,7,42,43]. Linoleic acid is a component of the cellular membrane structure [44]; it was chosen to test the capacity of the extracts to inhibit lipoperoxidation because it is an essential fatty acid afforded by food. To understand at what lipoperoxidation step the extracts could act, we designed a model including three major steps of lipid oxidation, beginning with short chain volatile C-compounds like pentane and ethylene, as these belong to the markers of an advanced lipid peroxidation, which results in breaking the C-chain [29]. In our model, their formation was analyzed by a gas chromatography (GC) technique by following the peak of ethylene production (Figure 1). Lipid hydroperoxides produced before the volatile compounds were analyzed by a spectroscopic technique using DMPD. This compound reacts with lipid hydroperoxides by forming a pink adduct [32], whose absorbance was measured at 517 nm. Thus, DMPD enables measuring the amount of lipid hydroperoxides produced, as illustrated on Figure 2. The initiation of a lipid peroxidation generates peroxyl and other transient free radicals, which were monitored by ESR technique. For all extracts, the data obtained with the DMPD technique (Figure 2) were in agreement with those from the measurement of ethylene (Figure 1) and evidenced a significant dose-dependent inhibitory effect, except at the concentration of 5 µg/mL, which was not applied in the ethylene measurement and achieved no significant effect *vs.* control in the spectroscopic assay with DMPD. 

With ESR technique, all extracts showed a quite similar inhibitory profile towards the production of intermediate peroxyl radicals (Figure 3A,B), except *Abelmoschus* and *Hibiscus* at 10 µg/mL concentration, which rather behaved as pro-oxidant (Figure 3B). These observations are almost identical for both boiled and not heated samples. *Manihot* and *Pteridium* had the best antiradical activity, hindering the formation of POBN/peroxyl radical adducts. 

For all three steps of lipoperoxidation, we observed the same AOX behavior, *i.e.*, a pronounced inhibition by *Manihot* followed by *Pteridium, Abelmoschus* and lastly *Hibiscus*. The results could be explained by the presence of polyphenol compounds within the extracts (Table 1, Figure 6A–D). Indeed, when reacting with free radicals, it is well known that polyphenols are readily transformed into stable phenoxyl radicals and thus, can terminate the chain of lipid peroxidation. On the other hand, the polyphenols contained in the vegetables could equally be considered as responsible for the AOX activity as they can react either directly with the ascorbyl radical, that triggers the lipid peroxidation, and thus decrease the peroxidation level already at its initiation, or with the lipid peroxyl radicals resulting from the attack of linoleic acid by the ascorbyl radical. Additionally to the GC and DMPD findings, the data obtained with ESR illustrate and underline the efficiency of the studied extracts to hinder the propagation of lipoperoxidation, by inhibiting the formation of transient free radicals at the start of lipid peroxidation called “initiation”, with a subsequent decrease of lipoperoxidation by-products such as lipid hydroperoxides and volatile compounds. 

Despite the difference in the methods used and in the amounts found, our findings are in agreement with those reported by investigations carried out on the presence of polyphenol and flavonoid in the vegetables that we tested and their AOX activities, particularly with regard to the variety *Hibiscus sabdariffa* and *Abelmoschus*, whereas those of *Manihot* and *Pteridium* remain poorly elucidated [19,20,21,22,23,24,25,26,27,28].

However, in the ESR-spin trap technique, a slight pro-oxidant effect was observed with *Abelmoschus* and *Hibiscus* at 5 µg/mL. It is well known that the same polyphenols that are well known to act efficiently against oxidation, can also be pro-oxidant, depending on their mechanisms of reaction and on the redox state of the system being studied [45,46]. 

To investigate the inhibitory potential of the selected vegetables towards ROS production in “inflammation like” conditions, like those obtained from monocytes activated with phorbol ester (PMA), we adapted a model based on the description of Amado *et al.* [35]. Monocytes have a low level of myeloperoxidase, a powerful oxidant enzyme shared by the defense cells. The ROS amount produced by monocytes is quite low, whereas the control is required to be high enough to efficiently monitor the AOX activities of the selected vegetables and allow comparisons. Therefore, we used HRP as reaction enhancer, because monocytes have “*per se*” a low ROS production and HRP enhances it. Our results show a significant decrease of fluorescence in a concentration-dependent manner, when the experiments were carried out in the presence of the extracts. This observation suggests that the extracts exert various AOX activities depending on their own polyphenol content, the mechanisms of reaction of their components, and the redox state of the reaction environment [10,11], particularly for *Manihot*. Similar results were obtained for both moderately boiled and not heated samples, with slightly more inhibitory effect for the not heated ones. 

Those findings are in agreement with literature data showing that some polyphenols contained in some fruit and vegetables act as efficient AOXs [10,11,19,20,21,22,23,24,25,47,48]. Our cellular results suggest that the extracts might act directly on reactive species such as H_2_O_2_, which results from O_2_^−^ dismutation and can cross the cell membrane [49,50]. Moreover, the extracts may also act on the cellular enzymes responsible for ROS production, mainly NADPH-oxidase which is the principal producer of superoxide anion (O_2_^−^). Other oxidant species like oxoferryl compounds generated by the reaction of peroxidase (HRP) and hydroxyl radical, which is not investigated in our study, could also have been scavenged by the polyphenols [10,11,49,50].

The effects of heat treatment on the AOX potential of polyphenols found in vegetal foodstuffs depend on the matrix of the foodstuff, the kind of the bonds between the antioxidant compounds and other components within the matrix, as well as on the level and duration of the heat treatment. The literature data reporting these effects still remain controversial [51,52,53]. The findings issued from our model of heat treatment encourage cooking the tested green vegetables with moderate heat treatment rather than a sudden aggressive one, because such moderate heat processing might preserve the AOX potential of the polyphenols contained in those plants. At the same concentration, the little differences of AOX activity among the plants might be explained by the profile of polyphenols contained in each plant, and their mechanisms of chemical action [10,11]. The HPLC-UV/DAD analysis identified some polyphenol compounds contained in the leaf powders, whose standards were available in the system, and which are well known to display AOX activities. However, some eluted compounds remained unidentified and need further analysis. 

## 5. Conclusion

The tested dietary plants were shown to contain polyphenols and flavonoids with well known AOX activities. In our lipid peroxidation model, the aqueous extracts of these plants significantly decreased the formation of typical markers of an advanced lipid peroxidation, such as lipid hydroperoxides and ethylene. Moreover, the extract solutions were shown to inhibit the formation of transient free radicals at an early stage of lipid peroxidation. 

In our cellular model, the same plant extracts developed no cell toxicity and decreased the ROS production by activating monocytes (HL-60 cells). A low heat treatment did not sensitively impair the AOX and antiradical potential that we observed.

Our data provide first insights into the AOX and antiradical properties of *Abelmoschus esculentus*, *Hibiscus acetosella*, *Manihot esculenta* and *Pteridium aquilinum* in a model of a complete lipid peroxidation, as well as under cellular “inflammation like” conditions. They evidence the importance of achieving deeper views into the profile of the AOX compounds contained in these plants, because they are eaten in the Philippines, Indonesia, Malaysia, other Asian countries and Latin America. Particularly for migrants from Western and Central Africa living in Western Europe and for more than 200 million consumers in sub-Saharan Africa, *Manihot* leaves are the basic green vegetable. Moreover, these dietary plants are equally utilized in folk medicine. Thus, the role of these plants in folk medicine by sub-Saharan peoples as well as in the prevention of oxidative stress and ROS related diseases is worthy of further consideration. 
